# The consequences of using statistical tests on proxy measurements in place of gold standard measurements: an application to magnetic resonance spectroscopy

**DOI:** 10.1038/s41598-025-32710-7

**Published:** 2025-12-25

**Authors:** Michael Treacy, Christoph Juchem, Karl Landheer

**Affiliations:** 1 Department of Radiology, Massachusetts General Hospital, Boston, USA; 2 High Field MR Center, Center for Medical Physics and Biomedical Engineering, Medical University of Vienna, Vienna, Austria; 3 Regeneron Genetics Center, LLC, 785 Old Saw Mill River Road, Tarrytown, NY USA

**Keywords:** Magnetic resonance spectroscopy, MEGA-edited GABA, Short-TE MRS, Hypothesis testing, Biochemistry, Chemistry

## Abstract

The use of proxy measurements in biomedical science is ubiquitous, due to the infeasibility or unavailability of gold-standard (i.e., most precise, accurate, and/or validated) measurements. For example, in magnetic resonance spectroscopy (MRS), short-echo time (TE) sequences are frequently employed to estimate difficult-to-measure metabolites such as GABA, despite J-difference editing being the recommended gold-standard due to improved metabolic specificity. This work investigates the critical relationship between the correlation of proxy and gold-standard measurements and the associated false positive (FPR) and false negative (FNR) rates of statistical tests performed on proxy measurements. Through statistical simulations, we demonstrate that even moderately high correlations (0.6–0.7), reported in the literature for short-TE vs. J-edited estimated GABA, can lead to drastically inflated FPRs and FNRs. We show that these rates are highly sensitive to the magnitude of differential bias in the proxy measurement (δ) and the underlying true effect size (Δ). For instance, a small, unmeasured bias in short-TE estimated GABA, potentially arising from macromolecule contamination, can substantially inflate FPRs. Conversely, imperfect correlation can substantially reduce statistical power, leading to high FNRs, which may explain some discrepancies within the literature. Although this work focuses specifically on the relationship between short-TE and MEGA-edited GABA, the arguments presented here apply more broadly to other difficult-to-measure metabolites in MRS (e.g., glutathione, 2-hydroxyglutarate), or generally to *any* circumstance where statistical tests are performed on the readily available proxy measurements in place of gold-standard measurements.

## Introduction

**Fig. 1:**
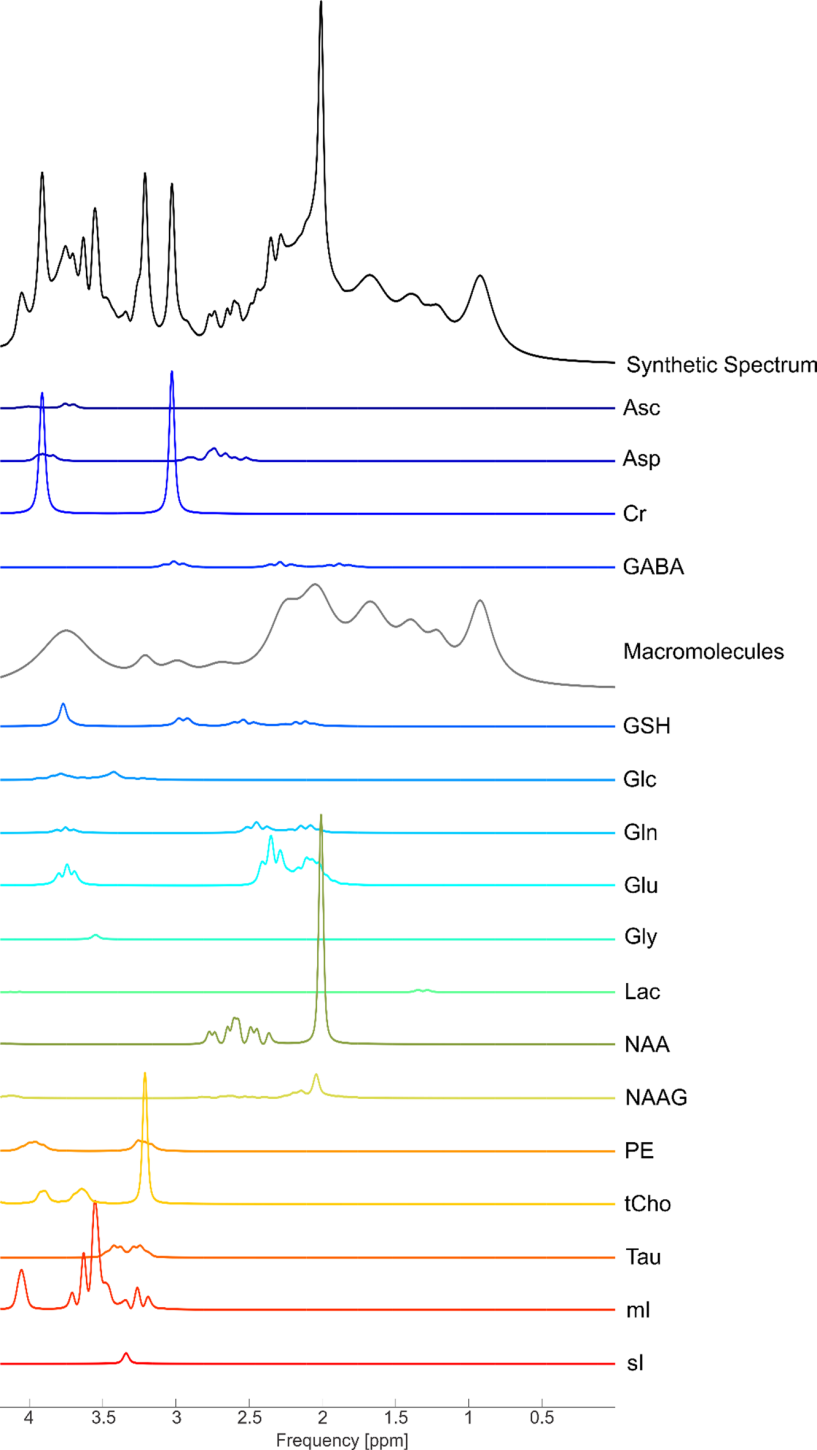
Synthetic spectrum of a typical short-TE (30 ms) PRESS sequence with repetition time = 2 s generated via synMARSS^49,50^ at 3T in the absence of noise with concentrations typical for healthy brain tissue. Note the low relative amplitude of difficult-to-quantify metabolites such as GABA and glutathione compared to numerous other components directly overlapping them, most notably the macromolecules. No individual moiety of either metabolite is free from spectral overlap with much more prominent resonances, which results in considerable quantification errors and moderate correlation with MEGA-edited quantification. Metabolites simulated include ascorbate (Asc), aspartate (Asp), creatine (Cr), γ-aminobutyric acid (GABA), macromolecules, glutathione (GSH), glucose (Glc), glutamine (Gln), glutamate (Glu), lactate (Lac), N-acetylaspartate (NAA), N-acetylaspartylglutamate (NAAG), phosphorylethanolamine (PE), total choline (tCho), taurine (Tau), myo-inositol (mI), scyllo-inositol (sI).

In biomedical science, proxy measurements are widely used in place of gold-standard measurements because the latter are often infeasible (due to resource or ethical constraints), impossible (due to inherent physical or technological limitations), or simply unavailable in existing datasets. In magnetic resonance spectroscopy (MRS), short-echo time (TE) sequences are appealing for several factors: wide availability (preinstalled stock PRESS^1^ and STEAM^2^ sequences on all major vendor platforms), high signal-to-noise ratio due to reduced T_2_-weighting^3^, reduced sensitivity to variations in T_2_ values, and easy quantification of typical major metabolites^3–5^ which may serve as a reference or may also be of interest. Short-TE sequences suffer from strong spectral overlap (Fig. 1), and as such J-editing modules such as MEGA^6^ have been developed to improve metabolic specificity^7–9^, and are the recommended standard^9,10^ for metabolites such as γ-aminobutyric acid (GABA), 2-hydroxyglutarate and glutathione. These metabolites are characterized by being low in amplitude and heavily overlapped with other signals, particularly macromolecules which have poorly characterized spectral shape^11^. Regardless, there are numerous examples in the literature of short-TE sequences being used to quantify GABA^12–22^, glutathione^13,15,17,23,24^ and 2-hydroxyglutarate^25–27^, to name a few.

Provided that the correlation is sufficiently high between the gold-standard measurement (the focus of this work will assume this is MEGA-edited GABA) and the proxy measurement (i.e., short-TE measured GABA), simpler approaches may be appropriate. We investigate what exactly constitutes a “sufficiently high correlation” here. This is achieved using Monte Carlo simulations, and we demonstrate that the false positive rate (FPR) and false negative rate (FNR) are strongly dependent on the correlation between measurements from MEGA- and short-TE estimate measurements. Even for the moderately-high correlations (0.6 to 0.7), representative of those from the literature for this application, the FPR and FNR can be drastically higher than reported, suggesting that results which rest on the assumption of perfect correlation need to be interpreted with increased caution^28,29^. A preliminary version of this work has previously been presented in abstract form^30^.

## Methods

**Fig. 2:**
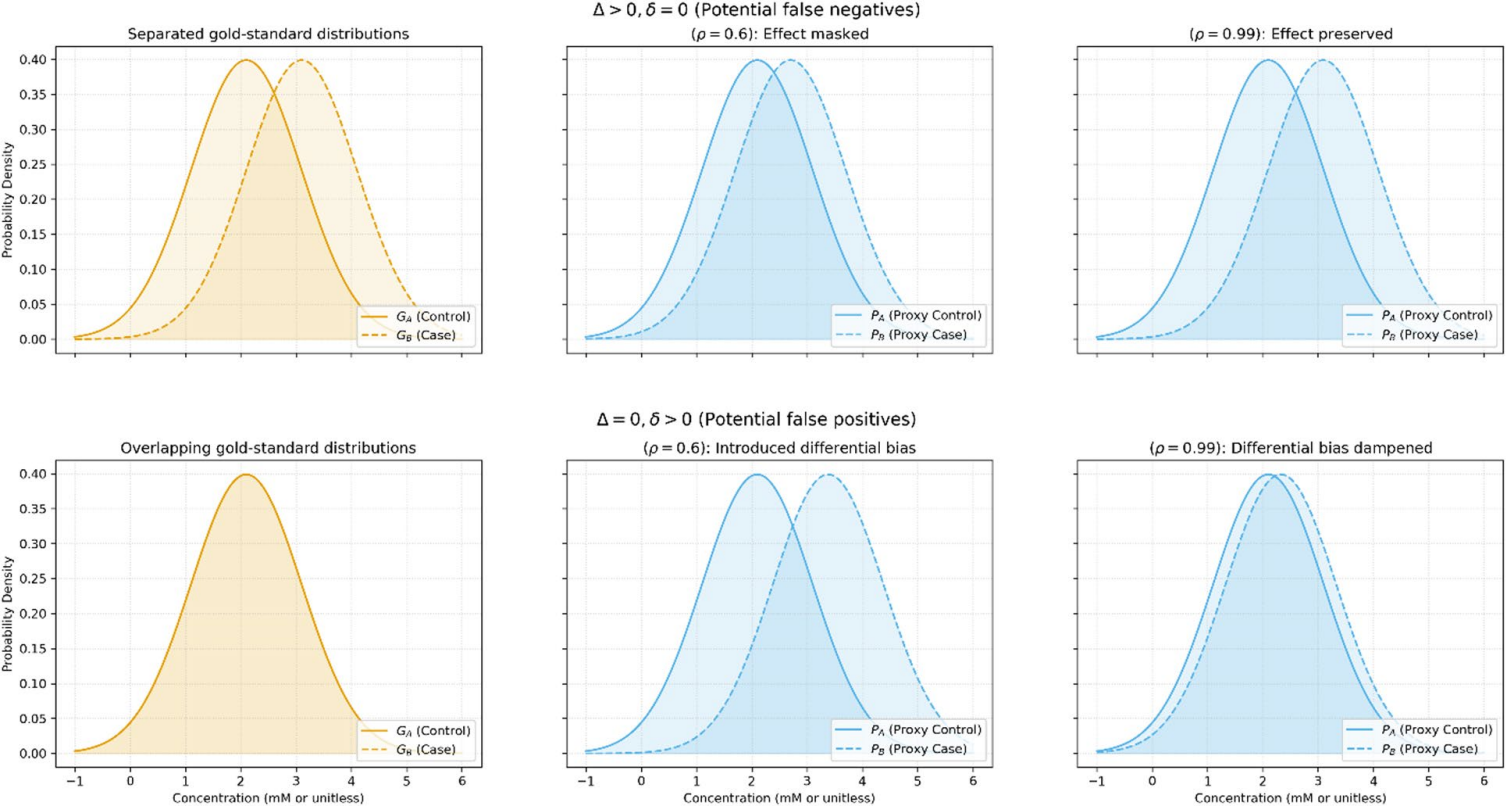
Conceptual diagram demonstrating the gold-standard distributions (left) and proxy distribution for a correlation of 0.6 (middle) and a near-perfect correlation of 0.99 (right). The case when $\Delta >0$ but $\delta =0$ is depicted in the top row, where false negatives may occur. In the top row it can be seen that for a moderate correlation there can be a substantial reduction in means (and hence a higher chance to obtain a false negative), which is resolved as the correlation approaches 1. Similarly, in the bottom row for a moderate correlation there can be a substantial difference in the mean of the proxy distributions, which is then resolved as the correlation approaches 1. The purpose of this work is to investigate the relationship at which a correlation becomes “sufficiently high enough” to not drastically inflate false positive and false negative rates.

We denoted ${G}_{A}$ and ${G}_{B}$ to be the random variables sampled from the gold-standard distributions (e.g., MEGA-edited measured concentrations for the metabolite of interest) of the control groups and cases, respectively. For example, ${G}_{B}$ could represent concentrations of GABA from subjects in a disease population^31^, or concentrations after a functional stimulus^19^. Similarly, we denoted ${P}_{A}$ and ${P}_{B}$ as proxy variables (i.e., short-TE measured GABA concentrations) of the control and case population, respectively. A conceptual diagram of this methodology is provided in Fig. 2 and a full derivation of all expressions and rationale for approximations are provided in the Appendix.

We model the gold-standard measurements by

1 $$G_{A} = S_{A} + {\epsilon}_{A} ,$$

2 $$G_{B} = S_{B} + {\epsilon}_{B} ,$$

where $S_{A} \sim N\left( {\mu_{bio} ,\sigma_{bio}^{2} } \right); {\epsilon}_{A} \sim N\left( {0,\sigma_{{G_{err} }}^{2} } \right);S_{B} \sim N\left( {\mu_{bio} + {\Delta },\sigma_{bio}^{2} } \right);{\epsilon}_{B} \sim N\left( {0,\sigma_{{G_{err} }}^{2} } \right)$, and $\sim N\left( {\mu , \sigma^{2} } \right)$ denotes sampling from the normal distribution with mean $\mu$ and standard deviation $\sigma$, both with units of millimole (mM) for concentrations, or unitless for relative concentrations. The true effect (i.e., differences in means between $G_{A}$ and $G_{B}$) is given by ${\Delta }$. $\mu_{bio}$ and $\sigma_{bio}^{2}$ represents the true mean and variance of the unobserved biological signal S, respectively. $\sigma_{{G_{err} }}^{2}$ is the non-biological measurement variance of the gold standard variables.

The proxy variables are then modeled as a linear combination of the true biological signal, S, and an independent noise/contamination component, $\zeta$

3 $$P_{A} = x_{1} S_{A} + x_{2} \zeta_{A} ,$$

4 $$P_{B} = x_{1} S_{B} + x_{2} \zeta_{B} ,$$

where $\zeta_{A} \sim N\left( {\mu_{contam} ,\sigma_{contam}^{2} } \right); \zeta_{B} \sim N\left( {\mu_{contam} + \delta ,\sigma_{contam}^{2} } \right).$ $\mu_{contam}$ and $\sigma_{contam}^{2}$ represent the mean and variance of the contamination signal $\zeta$ due to the use of proxy variables. $x_{1}$ is the sensitivity coefficient (i.e., how sensitive the proxy measurement is to the true signal, and $x_{2}$ is the contamination coefficient. $\delta$ represents *differential* bias introduced due to the use of proxy variables that is present only in cases and not controls (or vice versa). Non-zero differential bias could arise in scenarios where one is measuring a difference between cases and controls that is assumed to be due to a change in GABA concentration but is actually due to a different reason (e.g., macromolecule changes in cases and controls). Further exploration of potential causes of non-zero differential bias are given in the Discussion. In order to re-parameterize $x_{1}$ and $x_{2}$ in terms of the correlation between the proxy and gold-standard measurements, we must introduce the variance ratio, defined to be $VR \equiv \frac{{var\left( {P_{A} } \right)}}{{var\left( {G_{A} } \right)}} = \frac{{var\left( {P_{B} } \right)}}{{var\left( {G_{B} } \right)}} \approx x_{1}^{2} + x_{2}^{2} \frac{{\sigma_{contam}^{2} }}{{\sigma_{bio}^{2} }} = x_{1}^{2} + x_{2}^{2} R_{\sigma }$, where $R_{\sigma } \equiv \frac{{\sigma_{contam}^{2} }}{{\sigma_{bio}^{2} }}$ (see Appendix).

We calculated the false positive and false negative rates numerically by running Monte Carlo simulations, where random variables representing the concentrations of proxy variables were generated according to the model defined by Eqs. 1–4. All simulations were performed with 500,000 iterations per set of parameters with NumPy version 2.1.3 and all statistical tests were two-sided independent Welch’s t-tests performed via SciPy version 1.15.3. We performed statistical tests on the proxy variables (i.e., $P_{A}$ and $P_{B}$) to test hypotheses about the underlying gold-standard variables. Specifically, the null hypothesis ($H_{0}$) was that no true difference exists between the gold-standard means ($H_{0} :{\Delta } = 0$), and the alternative hypothesis ($H_{1}$) was that a true difference does exist ($H_{1} :{\Delta } \ne 0)$. The code to reproduce all statistical simulations is available at: github.com/karllandheer/GoldStandardVsProxy**.**

To investigate FPRs, we set ${\Delta } = 0$ (i.e., $H_{0}$ is true, no true difference exists between the means of the gold-standard variables) and $\delta > 0$, (i.e., a differential bias is introduced in the estimation of cases but not controls of the proxy variables, or vice versa). To investigate FNRs, we set $\delta = 0$ (no differential bias) but ${\Delta } > 0$ ($H_{1}$ is false). Initially, we array across a large range of possibilities of ${\Delta }$ or $\delta$ (from 0.2 to 2.2 mM) to demonstrate that in certain scenarios both FPR and FNR can be strongly sensitive to correlation and refer to these as the “theoretical simulations”. The parameter $\sigma_{bio}$ was set to 1 mM, and simulations are independent of the choice of $\mu_{bio}$ and $\mu_{contam}$ (as shown in the Appendix). We choose the nominal false-positive rate $\alpha = 0.05,$ and $N_{samples} \in \left\{ {10,{ }25, 100} \right\}$. We set VR = 1 (as the test statistic is independent of it if $\sigma_{bio}$ is fixed, as shown in the Appendix) and three values for $R_{\sigma }$ ∈ {0.5, 1, 2} when $\delta \ne 0$. For $\delta = 0$ the test statistic is independent of $R_{\sigma }$, hence we do not array over it. We also investigate how the false positive and false negative rate changes as we sweep across a range of $\alpha$ values.

We then performed statistical simulations with experimental values from the literature and refer to these as the “literature simulations”. First, to investigate the influence of correlation and the differential bias δ on the FPR, we used values from the literature which report statistically significant difference in the concentration of GABA in the visual cortex before and after behavioral training as assessed by short-TE MRS at 7T^19^. We assume the reported sample values are reflective of the population values, i.e., $\sigma_{P} = 1.55$, $E\left( {P_{A} } \right) = 6.8$, $E\left( {P_{B} } \right) = 7.6$. We use their values of N_samples_ = 25, $\alpha = 0.05$. If we assume the null hypothesis is correct (i.e., ${\Delta } = 0),$ then $\delta = \frac{{E\left( {P_{B} } \right) - E\left( {P_{A} } \right)}}{{\sqrt {1 - \rho^{2} } }}\sqrt {\frac{{R_{\sigma } }}{VR}} .$ As we only have empirical values for $E\left( {P_{B} } \right) - E\left( {P_{A} } \right)$ and $\rho$ (0.72 at 7T^32^), we thus must assume certain values for $R_{\sigma }$ and VR. If we set $R_{\sigma } = VR = 1$, then $\delta$ = 1.2. However, we investigate how sensitive these results are to $R_{\sigma }$ by also performing simulations with $R_{\sigma } = 0.5$ and $R_{\sigma } = 2,$ while keeping $\delta$ fixed, and since $\sigma_{bio}$ is not known, but rather $\sigma_{P}$, we must also simulate a range of variance ratios $VR$ ∈ {0.5, 1, $VR_{max}$} (as explained further in the derivation of the t-statistic section in the Appendix). We provide the mathematical context of how such a $\delta$ may arise due to small macromolecule contamination in the Discussion.

To investigate the influence of correlation on FNR, we then used values from the literature which used short-TE to estimate GABA at 3T but failed to find a difference between ALS subjects^12^. The relevant values from this study are $N_{samples} = 9/10$ (cases/controls) a correlation of 0.58 (previously measured at 3T^16^), $\sigma_{P} = 0.06$ and $\alpha = 0.05$. Previously, it was observed that ALS patients had a 16% reduction in GABA (1.42 in ALS patients vs 1.70 in healthy controls^33^), thus we assumed the true effect size is ${\Delta } = - 0.05$. Because we assume $\delta = 0$, the test statistic is independent of $R_{\sigma }$, and hence is not arrayed over, while $VR$ ∈ {0.5, 1, $VR_{max}$}.

## Results

**Fig. 3:**
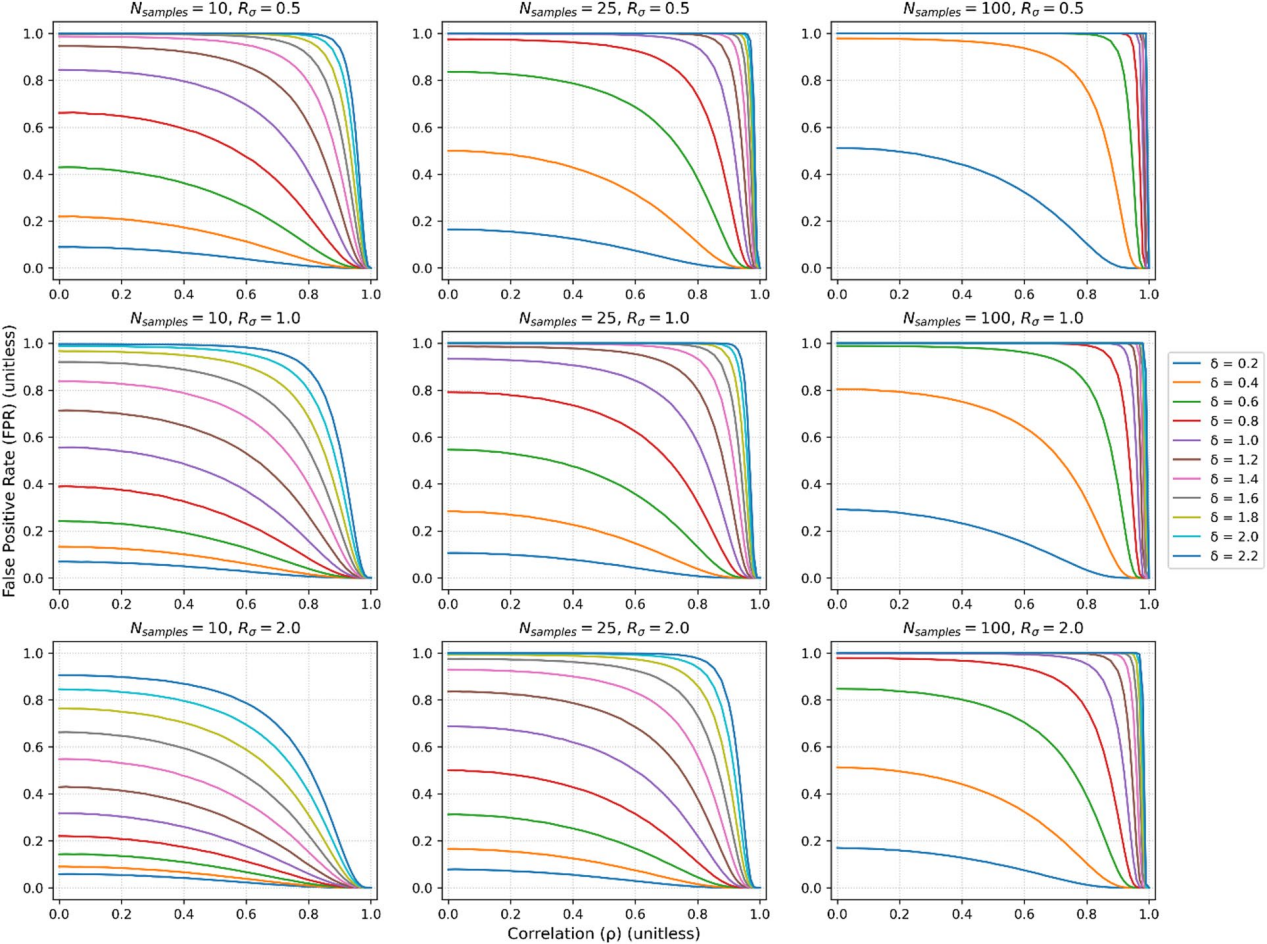
False positive rate vs correlation across a range of bias values ($\delta$). ${\sigma }_{bio}=1 \space \mathrm{mM}$, $\alpha =0.05$ and an array across a typical number of subjects within the neuroimaging literature, N_samples_ = 10 (left column), N_samples_ = 25 (middle column) and N_samples_ = 100 (right column). Values of ${R}_{\sigma }\equiv \frac{{\sigma }_{contam}^{2}}{{\sigma }_{bio}^{2}}$ were 0.5 (top row), 1 (middle row) and 2 (bottom row). At even moderate to strong correlations (0.4 to 0.7), provided $\delta$ is sufficiently large, the false-positive rate can be nearly 100%. The false positive rate *increases* with increasing sample size, as the experiment is better powered to detect a false result. Similarly, the false positive rate decreases with increasing ${R}_{\sigma }$, as the contaminating *signal* is drowned out by the contaminating *noise*.

In the case when $\Delta =0$, FPR increases rapidly with correlations that deviate from unity if the proxy-measurement bias, $\delta$, is large (Fig. 3). Even at moderate to strong correlations (0.4 to 0.7), provided $\delta$ is sufficiently large, the FPR can be ~ 100%. The false positive rate *increases* with increasing sample size, as the experiment is better powered to detect a false result. Importantly, the false positive rate decreases with increasing ${R}_{\sigma }$, which is expected as the greater noise from the use of the proxy variable drowns out the detected bias.

**Fig. 4:**
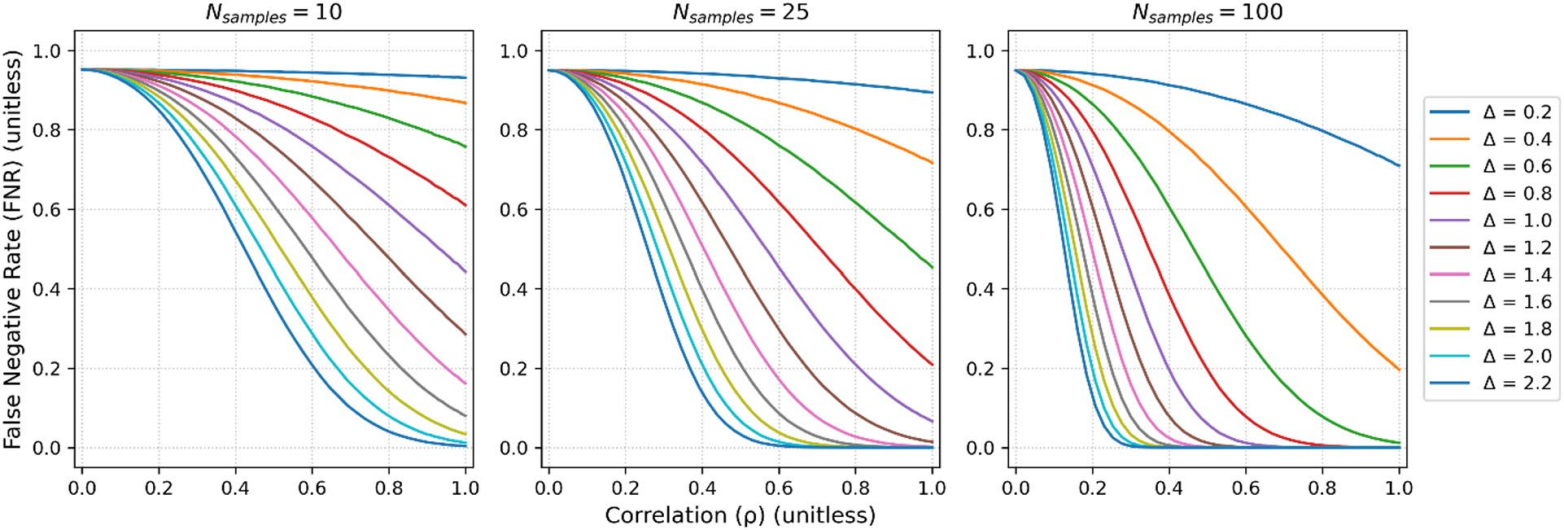
False negative rate vs correlation across a range of differences between gold standard measurements ($\Delta$). ${\sigma }_{bio}=1 \space \mathrm{mM}$, $\alpha =0.05$ and an array across a typical number of subjects within the neuroimaging literature, N_samples_ = 10 (left column), N_samples_ = 25 (middle column) and N_samples_ = 100 (right column). Values of ${R}_{\sigma }$ were not swept as the FNR is independent of it (see Appendix). Even with moderate-to-strong correlations the false negative rate can be substantially inflated. For example, with N_samples_ = 25 and $\Delta =1.0\space \mathrm{mM}$ at a correlation of 1 the false negative rate is approximately 7%, while at a correlation of 0.7 it’s approximately 33%. The false negative decreases with ${N}_{samples}$, as expected, as the experiment is better powered to detect the positive. At low correlations regardless of differences between the true distributions the false negative rate converges to the value of $1-\alpha$, as expected.

**Fig. 5:**
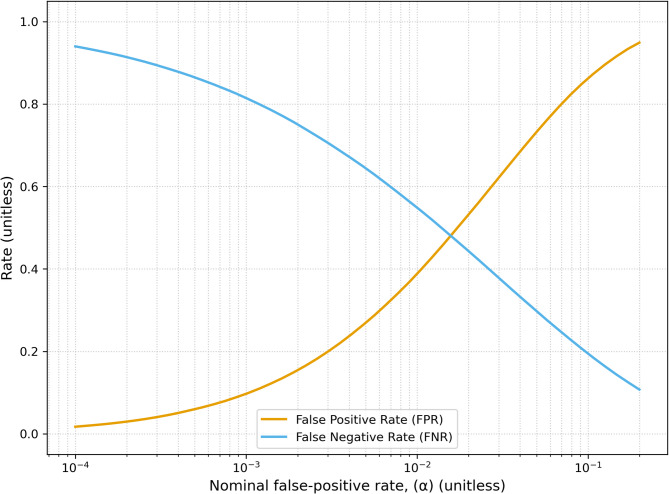
False positive rate (beige) and false negative rate (blue) vs nominal false-positive rate, $\alpha$. The simulations used moderate values of $\Delta =1$ mM, $\delta =0$ for the false negative rate, and $\Delta =0, \delta =1$ mM for the false positive rate, with ${\sigma }_{bio}=1$ mM and N = 25. A strategy to mitigate false positives may appear to decrease $\alpha$, however this comes at an increased false negative rate, which may or may not be equally undesirable.

In the case when $\Delta \ne 0$, but $\delta =0$, even with moderate to strong correlation between gold-standard and proxy measurements, the use of proxy measurements results in a substantial increase in FNR (Fig. 4). For example, with ${N}_{samples}$ = 25 and $\Delta =1.0 \space \mathrm{mM},$ the false negative rate can be ~ 7% at $\rho =1$, while at a $\rho =0.7$ it increases to ~ 33%. As the nominal false-positive rate is increased the measured false positive rate increases, while the false-negative rate decreases (Fig. 5). Thus, while reducing $\alpha$ is a viable strategy for reducing the false positive rate, it concomitantly comes with an increase in false negative rate, which may or may not be an acceptable trade-off.

**Fig. 6:**
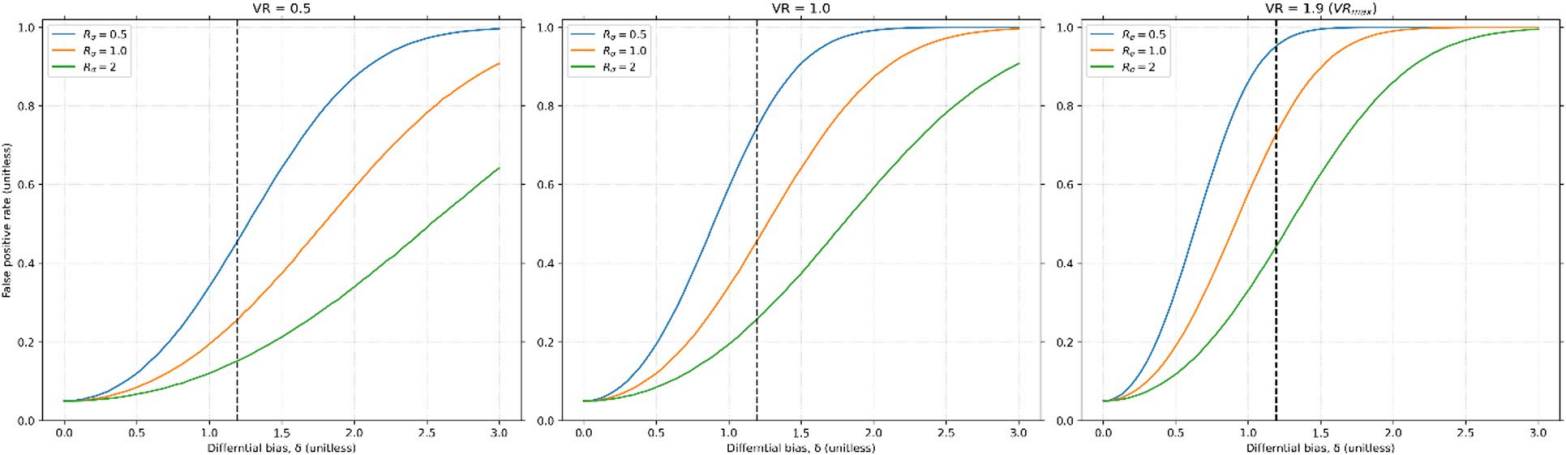
False positive rate versus $\delta$ with experimental values claiming to find a statistically significant difference in GABA/Creatine before and after behavioral training^19^. The dotted black line indicates the empirically estimated differential bias of $\delta =1.2$. Simulations were performed with values from their study^19^, specifically ${N}_{samples}=25$, $\alpha =0.05$, ${\sigma }_{P}=1.15$ and $\rho =0.72$*.* Three sets of ${R}_{\sigma }$ and $VR$ are depicted. The range of VRs is necessary because ${\sigma }_{P}$ is estimated, not ${\sigma }_{bio}$, and the range of ${R}_{\sigma }$ is necessary because the t-statistic is dependent on this value when $\delta \ne 0$. Even in the most optimistic scenario (VR = 0.5, ${R}_{\sigma }=0.5$), with $\delta =1.2$ the false positive rate is inflated to roughly three times its nominal value (15%) and can reach as high as 100% in other scenarios. While this range is large, it demonstrates that correlations alone cannot guarantee statistical validity of tests on proxy variables.

Using experimental values from the literature^19^*,* we demonstrate that the FPR increases dramatically with increasing magnitude of $\delta$. At the estimated $\delta =1.2$, the FPR ranges from 0.15 ($VR=0.5,{R}_{\sigma }=2)$ to 1.00 ($VR=1.9 (V{R}_{max}),{R}_{\sigma }=0.5)$, as shown in Fig. 6. When ${R}_{\sigma }$ is large, the high variance of the contaminant noise drowns out the differential bias, and when VR is small, the large biological variance ${\sigma }_{bio}$ also drowns out the bias. In other scenarios the differential bias can massively inflate the FPR, with it being as high as ~ 100%, indicating that their purported result should be interpreted with increased caution.

**Fig. 7:**
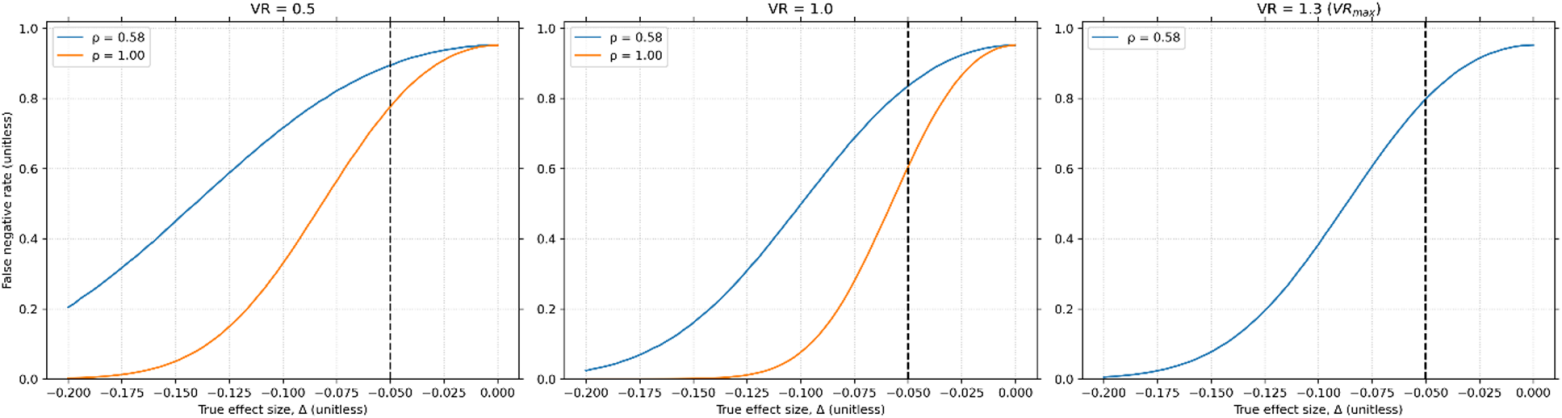
False negative rate versus $\Delta$ using experimental values in a study that disagreed with a previously reported significant difference in GABA/creatine between ALS patients and healthy controls^12^ for the empirical correlation at 3T (blue) and if the correlation was 1 (orange). Simulations were performed with values from their study, specifically ${N}_{samples}=9/10$ (cases/controls), $\alpha =0.05$ and ${\sigma }_{P}=0.06$*.* The dotted black line indicates the empirical $\Delta =-0.05$, obtained from a previous study^33^. The range of VRs is necessary because ${\sigma }_{P}$ is measured, not ${\sigma }_{bio}$, while a range of ${R}_{\sigma }$ is unnecessary because the t-statistic is independent on this value since $\delta =0$. The experimentally measured correlation of 0.58 drastically reduces the statistical power for all 3 VRs displayed here, although it is worth mentioning that for the previously measured $\Delta =-0.05$, the study is poorly powered even with $\rho =1$.

Using values from the literature^12^, we show that the use of proxy variables can also substantially increase the false negative rate (i.e., reduce statistical power). At the empirical correlation of 0.58 at 3T^16^, the previously measured $\Delta =-0.05$ results in a FNR of 0.89, 0.83, and 0.79 for VR = 0.5, 1 and 1.3 ($V{R}_{max}$), respectively. With a correlation of 1 the FNR is 0.77 and 0.59, for VR = 0.5 and 1, respectively (a correlation of 1 cannot be obtained for $V{R}_{max}$), Fig. 7). Thus, this apparent discrepancy between Blicher, et al.^12^ and Foerster, et al.^33^ may be explained by the study by Blicher et al. being simply underpowered, or due to the use of short-TE GABA which reduces statistical power.

## Discussion

This work suggests comprehensively characterizing the relationship between proxy and gold-standard methods is critical for sound inference of experiments using the former. We demonstrated via Monte Carlo simulations that typical correlations found in the literature between short-TE and MEGA-edited GABA do not preclude the possibility of drastically increased false positive and false negative rates. It is important to mention that the assumptions in our model (Appendix) are shown to be consistent with the literature, as they successfully reproduce the moderate correlations often cited as a basis for using short-TE measurements. We have shown across a wide range of parameters (ρ, VR, ${R}_{\sigma }$, N, Δ ,δ) that the use of imperfectly correlated proxy measurements can result in the substantial inflation of false positive and false negative rates. Because these critical parameters are not routinely measured, our goal is to highlight the need to quantify them and to caution against interpreting proxy-based tests as if they were gold-standard results.

It is important to note that while this work focused on the MEGA-edited experiments as the purported gold standard for certain metabolites^9^, MEGA-edited measurements are themselves a proxy for true concentrations. Thus, this work also has implications for the interpretation of MEGA-edited measurements. Currently, high-quality independent validation measurements of MEGA-edited MRS with other MR or non-MR methods are limited. Selected ex vivo validation of post-mortem tissues have been performed preclinically^34^ and clinically^35^. For example, in one animal study, the correlation of short-TE measured GABA with ex vivo concentrations from enzyme-linked immunosorbent assay was 0.66 at 7T^34^, once again in the range where FPRs and FNRs can be inflated. Statistical advancements on how to best combine proxy and gold-standard measurements are emerging^36^. Other typical quantified errors do not address the issue investigated here for two important reasons: firstly, and most importantly, we demonstrate that one can be measuring effects (e.g., macromolecules) entirely distinct from the effects one believes they are measuring (GABA), even while maintaining medium-to-high correlations with the desired quantity. Secondly, estimation of uncertainty is invariably difficult in MRS, as typical estimates via the Cramér-Rao Lower Bound require knowledge of the underlying model to fit the experimental data to, which is not known^37^, and distribution-free uncertainty estimation methods such as Conformal Predictors require access to underlying ground truth examples^38,39^, which are also inherently unavailable.

To estimate the increase in FPR over the nominal value the differential bias, $\delta$, must be known. While such values are experimentally unmeasured, the calculation below shows that a small variation in macromolecule concentration, due to their substantially higher amplitudes at short-TEs, could result in values comparable to those used in simulations in this work. GABA has a concentration of approximately 2.1 mM in the healthy brain^3,40^, and the overlying macromolecules have an absolute proton concentration, ${C}_{M,abs}$, of 19.8 mM at 2.99 ppm, 120.8 mM at 2.04 ppm, and 49.0 mM at 2.26 ppm, which nearly directly overlap with the GABA moieties at 3.01 ppm, 1.89 ppm and 2.23 ppm, respectively. Thus, using the institutional units where GABA/tCr $\cong$ 7.6 from Jia et al*.*^19^ (referred to as ${C}_{GABA,inst}$) and absolute concentration of GABA is $2.1$ mM (referred to as ${C}_{GABA,abs}$), the institutional concentrations of these three macromolecule resonances would be ${C}_{M,inst}={C}_{M,abs}\frac{{C}_{GABA,inst}}{{C}_{GABA,abs}}\frac{{N}_{p,M}}{{N}_{p},GABA}\left[\frac{\mathrm{exp}\left(-\frac{TE}{T{2}_{M}}\right)}{\mathrm{exp}\left(-\frac{TE}{T{2}_{GABA}}\right)}\right]$, where the $\frac{{N}_{p,M}}{{N}_{p},GABA}$ corrects for ${C}_{M,abs}$ being a proton concentration^11^, and ${C}_{GABA,abs}$ being a molecular concentration (this factor is equal to ½ for all resonances as all moieties of GABA contain 2 protons^41^) and the last term corrects for the difference in T_2_ values between GABA and macromolecules at the experimental TE of 36 ms. T_2_ values of 63 ms were used for GABA (measured at 7T^42^), and 20.0 ms, 14.3 ms, 19.8 ms, for M_2.99_, M_2.04_, M_2.26_, (measured at 3T^11^ with similar values obtained at 9.4T^43^). Thus, ${C}_{{M}_{2.99},inst}$, ${C}_{{M}_{2.04},inst}$, ${C}_{{M}_{2.26},inst}$ are 10.5, 31,2, and 25.5 (unitless), respectively. Thus, if even a small change (i.e., a few %) in these macromolecules concentrations between conditions bled into the GABA signal for cases but not controls (or vice versa), it could result in a $\delta$ comparable to the observed value of 1.2 (Fig. 6). While Jia et al*.*^19^ used an inversion preparation module to measure the macromolecules, this only partially alleviates the issue as the macromolecule measurement can vary substantially from those in the short-TE spectrum. This is because the inversion preparation module heavily weights the T_1_ spectrum^11^. Furthermore, although macromolecules are most problematic due to their poorly characterized spectral shape, there are numerous other directly overlapped metabolites with GABA, such as NAA, NAAG, GSH, glutamate (Glu) and glutamine (Gln) (Fig. 1) which could also potentially bleed into the estimated GABA quantity. While macromolecules are a likely contender for the introduction of such bias, other experimental differences between cases and controls could also cause it. Potential causes include differences in shim quality due to tissue heterogeneity differences^44^ (e.g., a tumor) or motion differences (which are known to be influenced by conditions such as Alzheimer’s^45^).

One limitation of the work presented here is the assumption of normality of distributions of the random variables. While this is a common assumption used within the biomedical literature (hence the use of t-tests), there is no inherent physiological reason why such concentrations would be sampled from a normal distribution. We chose the normal distribution as, for a given mean and standard deviation, it maximizes the entropy of the random variables. Hence in the absence of additional information about the distributions it becomes a natural choice. It is also worth mentioning that while the literature simulations focused specifically on GABA/tCr, if GABA and tCr are both sampled from normal distributions and $\frac{{\sigma }_{tCr}}{{\mu }_{tCr}}\ll 1$ (which has previously been reported as 0.06 at 3T^46^), then this ratio will also sample from a normal distribution. Work on characterizing the distributions of metabolite concentrations from both healthy and diseased populations would be a valuable contribution and could be used to further refine the results obtained here, among other simulations. Regardless, while the exact numerical results will invariably change based on choice of distributions, the problem investigated here will persist. Importantly, the Jia et al.^19^ study investigated GABA/tCr, and previous related studies have demonstrated that spurious correlations can arise due to the use of a common denominator^47^, thus further complicating the interpretation of such results.

## Data Availability

The code to generate all data and reproduce all results is available at: github.com/karllandheer/GoldStandardVsProxy.

## Appendix

### Definition of model

1. The True Biological Signal (S): This is the true, unobserved biophysical quantity of interest (e.g., GABA concentration). It is a random variable with a mean $E\left( S \right) = \mu_{bio}$ and variance $var\left( S \right) = \sigma_{bio}^{2}$.

2. The Gold-Standard Measurement (G): This is the most precise, accurate and/or validated measurement of a quantity of interest (in the context of this work, it is MEGA-edited GABA). We denote $G_{A}$ and $G_{B}$ as the gold-standard measurements for the controls and cases in Eqs. 1 and 2. The gold-standard measurement represented by

$$G = S + {\epsilon},$$

where ${\epsilon}$ is measurement error, assumed to be sampled from a zero-mean normal distribution; $E\left( {{\epsilon}} \right) = 0$ and $var\left( {{\epsilon}} \right) = \sigma_{{G_{err} }}^{2}$. Note, while gold-standard measurements will inherently possess a constant systematic bias relative to absolute biological truth (i.e., $E\left( {{\epsilon}} \right) \ne 0$), such a constant cancels out in the calculation of the effect size (${\Delta }$, the difference in means) and does not affect the derived false positive or false negative rates, hence was set to 0 here for simplicity. Thus $var\left( G \right) = var\left( S \right) + var\left( {{\epsilon}} \right) = \sigma_{bio}^{2} + \sigma_{{G_{err} }}^{2}$. We make the simplifying assumption that $\sigma_{{G_{err} }}^{2} \ll \sigma_{bio}^{2}$, i.e., the gold-standard measurement variance is dominated by the biological variance. Previous methods have found GABA + /water intra-class correlation $ICC \equiv \frac{{\sigma_{bio}^{2} }}{{\sigma_{bio}^{2} + \sigma_{{G_{err} }}^{2} }} \cong$ 0.87^48^. From this one can show that $\frac{{\sigma_{Gerr}^{2} }}{{\sigma_{bio}^{2} }} = \left( {1 - ICC} \right)/ICC \approx 0.15$. Indicating that $\sigma_{bio}^{2}$ is approximately 7 × larger than $\sigma_{Gerr}^{2}$.

3. The Proxy Measurement (P): This variable represents the measurement obtained from the proxy method (e.g., short-TE GABA), which we denote by $P_{A}$ and $P_{B}$ for controls and cases, as in Eqs. 3–4. We model $P$ as a weighted sum of the true signal S and an independent contamination/noise component $\zeta$

$$P = x_{1} S + x_{2} \zeta ,$$

where $\zeta$ is the contaminating signal (e.g., overlapping macromolecules or other metabolites), $x_{1}$ is the sensitivity coefficient (i.e., how sensitive the proxy measurement is to the true signal, and $x_{2}$ is the contamination coefficient. Because $\zeta$ and ${\epsilon}$ are noise terms, we assume S, ${\epsilon}$ and $\zeta$ are all mutually independent.

### Derivation of proxy variance and correlation

The variance of the proxy variable can be obtained from its definition

$$\begin{aligned} var\left( P \right) & = var\left( {x_{1} S + x_{2} \zeta } \right) = x_{1}^{2} var\left( S \right) + x_{2}^{2} var\left( \zeta \right) \\ & = x_{1}^{2} \sigma_{bio}^{2} + x_{2}^{2} \sigma_{contam}^{2} . \\ \end{aligned}$$

The correlation between the gold-standard and proxy variable is

$$\rho \equiv { }\frac{{cov\left( {G,P} \right)}}{{\sqrt {var\left( G \right)var\left( P \right)} }} .$$

The covariance term can be expressed as $cov\left( {G,P} \right) = cov\left( {S + {\epsilon},x_{1} S + x_{2} \zeta } \right) = x_{1} cov\left( {S,S} \right) = x_{1} \sigma_{bio}^{2}$, which rests on the mutual independence assumption of S, $\zeta$ and ${\epsilon}$ and the bilinearity of covariance. Thus, the correlation becomes

$$\begin{aligned} \rho & = \frac{{x_{1} \sigma_{bio}^{2} }}{{\sqrt {\left( {\sigma_{bio}^{2} + \sigma_{{G_{err} }}^{2} } \right)\left( {x_{1}^{2} \sigma_{bio}^{2} + x_{2}^{2} \sigma_{contam}^{2} } \right)} }} \\ & \approx \frac{{x_{1} \sigma_{bio}^{2} }}{{\sqrt {\sigma_{bio}^{2} \left( {x_{1}^{2} \sigma_{bio}^{2} + x_{2}^{2} \sigma_{contam}^{2} } \right)} }} \\ & = \frac{{x_{1} }}{{\sqrt {x_{1}^{2} + \frac{{x_{2}^{2} \sigma_{contam}^{2} }}{{\sigma_{bio}^{2} }}} }} \\ & = \frac{{x_{1} }}{{\sqrt {x_{1}^{2} + x_{2}^{2} R_{\sigma } } }}, \\ \end{aligned}$$

where we have defined $R_{\sigma } \equiv \frac{{\sigma_{contam}^{2} }}{{\sigma_{bio}^{2} }}$, and the approximation arises due to the assumption of $\sigma_{{G_{err} }}^{2} \ll \sigma_{bio}^{2}$. We then define the variance ratio, VR, as

$$VR \equiv \frac{var\left( P \right)}{{var\left( G \right)}} = \frac{{x_{1}^{2} \sigma_{bio}^{2} + x_{2}^{2} \sigma_{contam}^{2} }}{{\sigma_{bio}^{2} + \sigma_{{G_{err} }}^{2} }} \approx x_{1}^{2} + x_{2}^{2} \frac{{\sigma_{contam}^{2} }}{{\sigma_{bio}^{2} }} = x_{1}^{2} + x_{2}^{2} R_{\sigma }$$

Combining the above two expressions we obtain $\rho = \frac{{x_{1} }}{{\sqrt {VR} }}$. Since we assume $x_{1} \le 1$ (as $x_{1} > 1$ would imply the proxy variable is *more* sensitive to the true signal than the gold standard), we note that the VR provides an upper bound for the attainable correlation, i.e., $\rho \le \frac{1}{{\sqrt {VR} }}.$ Also, from these expressions it can be shown that $x_{1} = \rho \sqrt {VR}$ and $x_{2} = \sqrt {\frac{{VR\left( {1 - \rho^{2} } \right)}}{{R_{\sigma } }}} .$

### Derivation of expectation values

The expectation value for the gold-standard control group is

$$E\left( {G_{A} } \right) = E\left( {S_{A} + {\epsilon}_{A} } \right) = \mu_{bio} .$$

And similarly, the expectation value for the gold-standard case group is

$$E\left( {G_{B} } \right) = E\left( {S_{B} + {\epsilon}_{B} } \right) = \mu_{bio} + {\Delta },$$

where ${\Delta }$ is the true desired effect size (i.e., $E\left( {G_{B} } \right) - E\left( {G_{A} } \right) = {\Delta }$). The expectation value for the proxy control group can be expressed as

$$E\left( {P_{A} } \right) = E\left( {x_{1} S_{A} + x_{2} \zeta_{A} } \right) = x_{1} \mu_{bio} + x_{2} \mu_{contam}$$

And similarly, the expectation value for the proxy case group can be expressed as

$$E\left( {P_{B} } \right) = E\left( {x_{1} S_{B} + x_{2} \zeta_{B} } \right) = x_{1} \left( {\mu_{bio} + {\Delta }} \right) + x_{2} \left( {\mu_{contam} + \delta } \right).$$

### Derivation of t-statistic

The t-statistic for a two-sample Welch’s t-test is the ratio of the true difference in means to the standard error of that difference:

$$t = \frac{{E\left( {P_{B} } \right) - E\left( {P_{A} } \right)}}{{\sqrt {\frac{{var\left( {P_{B} } \right)}}{{n_{B} }} + \frac{{var\left( {P_{A} } \right)}}{{n_{A} }} } }} ,$$

where $n_{A}$ and $n_{B}$ are the sample sizes of the control and case groups, respectively. Given that $var\left( {P_{A} } \right) = var\left( {P_{B} } \right) = var\left( P \right)$ and that $var\left( P \right) = VR \cdot var\left( G \right) = VR\left( {\sigma_{bio}^{2} + \sigma_{{G_{err} }}^{2} } \right) \approx \sigma_{bio}^{2} VR$ and that $E\left( {P_{A} } \right) - E\left( {P_{B} } \right) = x_{1} {\Delta } + x_{2} \delta$ this becomes

$$t \approx \frac{{x_{1} {\Delta } + x_{2} \delta { }}}{{\sqrt {\frac{{\sigma_{bio}^{2} VR}}{{n_{B} }} + \frac{{\sigma_{bio}^{2} VR}}{{n_{A} }} } }} = \frac{{\rho \sqrt {VR} {\Delta } + \sqrt {\frac{{VR\left( {1 - \rho^{2} } \right)}}{{R_{\sigma } }}} \delta { }}}{{\sigma_{bio} \sqrt {VR} \sqrt {\frac{1}{{n_{B} }} + \frac{1}{{n_{A} }}} }} = \frac{{\rho {\Delta } + \sqrt {\frac{{\left( {1 - \rho^{2} } \right)}}{{R_{\sigma } }}} \delta { }}}{{\sigma_{bio} \sqrt {\frac{1}{{n_{B} }} + \frac{1}{{n_{A} }}} }}$$

which is independent of parameters $\mu_{contam}$ (introduction of bias in both cases and controls from use of proxy variable) and $\mu_{bio}$ (the true mean of the biological signal). Furthermore, if we assume $\sigma_{bio}$ is a known constant then the t-statistic is independent of VR and independent of $R_{\sigma }$ when $\delta$ = 0, but proportional to $1/\sqrt {R_{\sigma } }$ if $\delta \ne 0$. In the fixed $\sigma_{bio}$ scenario the only effect VR has on these simulations is that it determines the upper bound for correlation, $\rho \le \frac{1}{{\sqrt {VR} }},$ if $x_{1} \le 1$. For this reason, we set VR = 1 for the theoretical simulations, as the purpose of this is to investigate the question of given a fixed biological signal, how does the use of proxy measurements distort the statistical results. Alternatively, we can express the t-statistic as

$$t = \frac{{\rho {\Delta } + \sqrt {\frac{{\left( {1 - \rho^{2} } \right)}}{{R_{\sigma } }}} \delta { }}}{{\sigma_{P} \sqrt {\frac{1}{VR}\left( {\frac{1}{{n_{B} }} + \frac{1}{{n_{A} }}} \right)} }},$$

which is proportional to $\sqrt {VR}$. Thus, if t is parameterized by $\sigma_{P} ,$ it is dependent on VR. In the literature simulations we choose this form, as $\sigma_{P}$ is measured.
